# Low hemoglobin is associated with worse outcomes via larger hematoma volume in intracerebral hemorrhage due to systemic disease

**DOI:** 10.1002/mco2.96

**Published:** 2022-02-23

**Authors:** Shuting Zhang, Yang Shu, Yunlong Chen, Xiaoyang Liu, Yu Liu, Yajun Cheng, Bo Wu, Peng Lei, Ming Liu

**Affiliations:** ^1^ Department of Neurology, West China Hospital Sichuan University Chengdu P. R. China; ^2^ State Key Laboratory of Biotherapy, West China Hospital Sichuan University Chengdu P. R. China; ^3^ West China School of Medicine Sichuan University Chengdu P. R. China

**Keywords:** consensus cluster, death, hemoglobin, ICH subtype, intracerebral hemorrhage

## Abstract

Whether hemoglobin is associated with outcomes of a specific subtype of intracerebral hemorrhage (ICH) is unknown. A total of 4643 patients with ICH from a multicenter cohort were included in the analysis (64.0% male; mean age [SD], 58.3 [15.2] year), of whom 1319 (28.4%) had anemia on admission. The unsupervised consensus cluster method was employed to classify the patients into three clusters. The patients of cluster 3 were characterized by a high frequency of anemia (85.3%) and mainly composed of patients of systemic disease ICH subtype (SD‐ICH; 90.0%) according to the SMASH‐U etiologies. In SD‐ICH, a strong interaction effect was observed between anemia and 3‐month death (adjusted odds ratio [aOR] 4.33, 95% confidence interval [CI] 1.60–11.9, *p *= 0.004), and the hemoglobin levels were linearly associated with 3‐month death (aOR 0.75, 95% CI 0.60–0.92; *p *= 0.009), which was partially mediated by larger baseline hematoma volume (*p *= 0.008). This study demonstrated a strong linear association between low hemoglobin levels and worse outcomes in SD‐ICH, suggesting that hemoglobin‐elevating therapy might be extensively needed in a specific subtype of ICH.

## Graphical abstract

In this paper, we have analyzed 4643 ICH patients in a prospective multicenter cohort study. We employed both the unsupervised consensus cluster method and the SMASH‐U etiologies scheme to classify the ICH patients. We found that, in both statistical and clinical classification system, low hemoglobin levels were linearly and consistently associated with 1‐month survival (adjusted hazard ratio [HR] 7.93, 95% CI 2.20–28.60, *p *= 0.002) and 3‐month death (aOR 0.75, 95% CI 0.60–0.92; *p *= 0.009) in patients of SD‐ICH subtype. Moreover, the mediation analysis revealed that associations between lower hemoglobin with poor outcomes were partially mediated by baseline hematoma volume (*p *= 0.008).

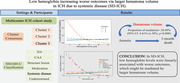

## 1 INTRODUCTION

Intracerebral hemorrhage (ICH) causes catastrophic brain damage, and there remain few therapeutic interventions that mitigate its severity. It accounts for around 25% of total strokes based on a national community‐based study in China, which is significantly higher than the 8%–15% in the Western populations.^1^, ^2^, ^3^ Beyond the risk factors of age, the severity of neurological impairment, and hematoma volume post‐ICH, few clinical biomarkers are available to predict outcomes in the long term.^4^, ^5^ In addition, most of these risk factors were not intervenable in clinical management post‐ICH.^6^

ICH is a group of heterogeneous cerebrovascular events of different etiologies with possible differential demands of hemoglobin.^7^ Hemoglobin is vital for oxygen supply and autoregulation of small arteries in essential organs such as the brain.^8^ Anemia (low blood hemoglobin levels) is a clinical determinant frequently reported to be associated with 3‐month death in critically ill patients, and a modifiable risk factor that could be treated, for example, with iron supplements or packed red cells transfusion.^9^, ^10^, ^11^, ^12^, ^13^ However, meta‐analysis has reported a high heterogeneity of studies regarding the association between ICH outcomes and hemoglobin, preventing definitive conclusions and developing subsequent treatment strategies.^14^

The variance may relate to the heterogeneity of the ICH patients of different natural histories and associated risk factors, but there is little knowledge of which factors could define the subgroup with a specific demand for hemoglobin. We hypothesized that different ICH subgroup patients have specific demands for hemoglobin. Here, in a large prospective multicenter cohort study of patients with ICH, we investigated whether ICH patients have different demands of hemoglobin using the unsupervised consensus cluster method and further explore its clinical significance.

## 2 RESULTS

### 2.1 Anemia is not associated with clinical outcomes in the general ICH cohort

**FIGURE 1:**
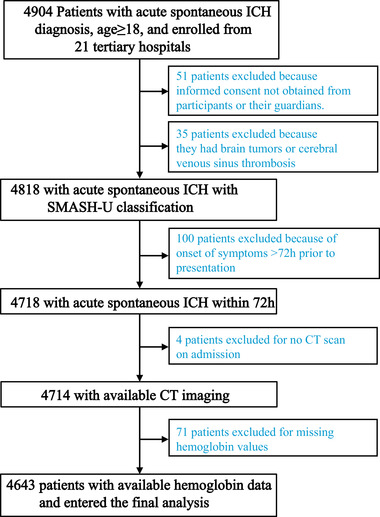
Flowchart of patients screened, included, and excluded from the study. Abbreviations: CT, computed tomography; ICH, intracerebral hemorrhage; SMASH‐U classification, an etiological classification of intracerebral hemorrhage.

In a multicenter cohort of spontaneous ICH subjects, we investigated whether clinical anemia or related hematological parameters were associated with functional outcomes over three months. A total of 4904 patients with spontaneous ICH were screened, and 4643 patients were included in the final analysis (Figure 1). Of 4643 patients, 2972 (64.0%) were male, and the mean age was 58.3 (±15.2) years. The mean hemoglobin concentration on admission was 13.5 (±2.0) g/dl, and anemia was diagnosed in 1319 patients (28.4%) (Table 1). Although the death at both 1 and 3 months post‐ICH was significantly more frequent in anemia patients than in nonanemia patients (11.1% vs. 7.7% and 23.0% vs. 17.4%, respectively; all *p *< 0.001) in univariable analysis, anemia was not associated with worse outcomes in both genders in multivariable regression. Moreover, there is no significant association between moderate anemia and a 3‐month death in the multivariate analysis for both genders (*p *= 0.310 and *p *= 0.106, respectively; Table S1).

**TABLE 1:** Baseline characteristics of the study population, stratified by anemia status

	**Total**	**Non‐anemia**	**Anemia**	
**Characteristics**	***n* = 4643**	***n* = 3324**	***n* = 1319**	***p***
Age (years)	58.3 ± 15.2	56.8 ± 14.7	61.9 ± 15.9	<0.001
Male, *n* (%)	2,972 (64.0%)	2,201 (66.2%)	771 (58.5%)	<0.001
Comorbidities and risk factors, *n* (%)				
HD history	319 (6.9%)	188 (5.7%)	131 (9.9%)	<0.001
Hypertension	2,619 (56.4%)	1,898 (57.1%)	721 (54.7%)	0.14
Diabetes mellitus	392 (8.4%)	250 (7.5%)	142 (10.8%)	<0.001
Chronic kidney disease	236 (5.1%)	95 (2.9%)	141 (10.7%)	<0.001
Anti‐thrombotics	406 (8.7%)	204 (6.1%)	202 (15.3%)	<0.001
Alcohol	918 (19.8%)	670 (20.2%)	248 (18.8%)	0.315
Smoking	1,160 (25.0%)	849 (25.5%)	311 (23.6%)	0.175
Clinical status				
GCS	13.0 (8.0, 15.0)	13.0 (8.0, 15.0)	13.0 (8.0, 15.0)	0.009
NIHSS	8.0 (3.0, 16.0)	8.0 (3.0, 16.0)	9.0 (4.0, 18.0)	0.001
Hematoma volume (ml)	12.7 (5.1, 27.0)	12.1 (5.0, 26.0)	14.0 (5.7, 30.0)	0.006
SBP (mm Hg)	162.3 ± 31.4	162.9 ± 31.3	160.7 ± 31.6	0.032
DBP (mm Hg)	95.0 ± 18.1	96.2 ± 18.2	91.9 ± 17.5	<0.001
Hemoglobin (g/dl)	13.5 ± 2.0	14.4 ± 1.5	11.3 ± 1.3	<0.001
HCT	0.41 ± 0.06	0.43 ± 0.05	0.35 ± 0.04	<0.001
Albumin (g/L)	41.4 ± 5.4	42.6 ± 5.0	38.6 ± 5.4	<0.001
Platelet count (10^9^/L)	158 (118, 204)	163 (125, 207)	142 (105, 191)	<0.001
PT, s	11.9 (11.1, 13.0)	11.8 (11.1, 12.8)	12.2 (11.4, 13.5)	<0.001
APTT, s	26.1 (23.2, 29.8)	25.7 (22.8, 29.3)	27.0 (24.1, 30.9)	<0.001
Fibrinogen (g/L)	2.9 (2.3, 3.6)	2.9 (2.3, 3.5)	2.9 (2.4, 3.7)	<0.001
INR	1.03 (0.97, 1.11)	1.02 (0.96, 1.09)	1.06 (0.99, 1.14)	<0.001
Blood glucose (mmol/L)	7.3 (6.1, 9.4)	7.4 (6.1, 9.4)	7.2 (6.0, 9.2)	0.048
Creatinine (μmol/L)	71.0 (58.0, 86.4)	71.0 (59.0, 85.0)	71.0 (56.0, 90.1)	<0.001
Hematoma Location, *n* (%)				
BG or thalamus	2,719 (63.4%)	1,958 (63.5%)	761 (63.1%)	0.819
Lobar	1,488 (33.3%)	1,018 (31.9%)	470 (36.9%)	0.001
Brainstem	373 (8.4%)	291 (9.1%)	82 (6.4%)	0.003
Cerebellar	275 (6.2%)	207 (6.5%)	68 (5.3%)	0.164
IVH	1,540 (33.2%)	1,083 (32.6%)	457 (34.6%)	0.379
SAH	360 (7.8%)	250 (7.5%)	110 (8.3%)	0.188
Therapies and complications, *n* (%)				
Anti‐hypertension	2,578 (59.0%)	1,902 (60.7%)	676 (54.6%)	<0.001
Dehydration	3,879 (88.7%)	2,795 (89.2%)	1,084 (87.5%)	0.116
Surgical intervention, *n* (%)	1,218 (26.2%)	893 (26.9%)	325 (24.6%)	0.129
Respiratory infection	1,007 (21.7%)	648 (19.5%)	359 (27.2%)	<0.001

*Notes*: Descriptive statistics were calculated using mean ± SD or median (IQR) for continuous variables and frequencies for categorical variables. Abbreviations: APTT, activated partial thromboplastin time; CAA, cerebral amyloid angiopathy; DBP, diastolic blood pressure; GCS, Glasgow Coma Scale; HA, hypertensive angiopathy; HCT, hematocrit; HD, heart disease; INR, international normalized ratio; IQR, interquartile range; IVH, intraventricular hemorrhage; NIHSS, National Institutes of Health Stroke Scale; PT, prothrombin time; SAH, subarachnoid hemorrhage; SBP, systolic blood pressure; SD, standard deviation.

### 2.2 Unsupervised consensus clustering identified a cluster where lower hemoglobin is associated with worse outcomes

**FIGURE 2:**
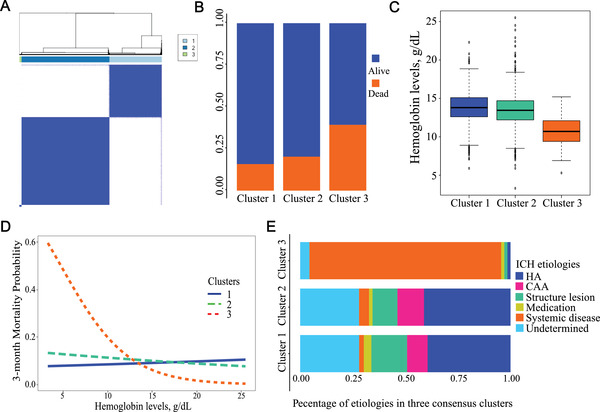
Characterization of ICH patients according to three consensus clusters divided using unsupervised consensus clustering. (A) Consensus values heatmap, demonstrating a delineated block structure for *k* = 3, supporting a three‐cluster solution; (B) 3‐month death rates in three consensus clusters; (C) the median of hemoglobin levels in three consensus clusters; (D) predicted outcome probabilities by hemoglobin levels in three consensus clusters; (E) percentage of the six ICH SMASH‐U etiologies in three consensus clusters. Abbreviations: CAA, cerebral amyloid angiopathy; HA, hypertensive angiopathy; ICH, intracerebral hemorrhage.

We hypothesized that the lack of association between anemia and clinical outcomes might be due to the high heterogeneity of the population. The consensus cluster method was employed to categorize unbiasedly the patients based on hemoglobin levels on admission and other standard variables, which might be associated with the clinical outcomes such as age, hematoma volume, blood glucose, and urea nitrogen levels (Figure 2A and Figure S1). The cohort could be categorized into three clusters, where the patients in cluster 3 have exhibited significantly higher death 3‐months post‐ICH (39.3% vs. 15.9% or 20.3%, respectively for clusters 3, 1 and 2, *p *< 0.001). Interestingly, compared to the other two clusters, cluster 3 was characterized by significantly lower hemoglobin levels (10.7 [2.0] vs. 13.8 [2.0] or 13.4 [2.0] g/dl, *p *< 0.001) and higher incidence of anemia (85.3% vs. 30.8% or 22.2%; Figure 2B,C and Table S2). There was a trend to indicate that the lower hemoglobin level was a risk factor for a 3‐month death in cluster 3 but not 1 or 2 (adjusted odds ratio [aOR] 0.76, 95% confidence interval [CI] 0.47–1.15, *p *= 0.222; Figure 2D).

To further understand the clinical nature of these clusters, we have compared them with an etiological classification method in clinical practice—the SMASH‐U scheme.^7^ We found that the patients of systemic diseases ICH subtype (SD‐ICH) account for 90.0% of the population in cluster 3 (Figure 2E), strongly suggesting that the hemoglobin levels may influence the clinical outcomes of SD‐ICH. While clusters 1 and 2 have shown no differences in their composition in ICH SMASH‐U classification, nor differences in hemoglobin levels (Figure 2C) or death 3‐months post‐ICH (Figure 2B). Collectively, these data implicated that the effect of low hemoglobin levels (or anemia) on clinical outcomes of ICH might be differentiated using clinical etiological classification.

### 2.3 Anemia interacted with systemic disease ICH subtype (SD‐ICH)

To test whether anemia contributes to the worse clinical outcome specifically in patients of SD‐ICH, we have evaluated the interaction effect between anemia and ICH subtypes (Table 2). When the interaction term of anemia × subtypes was introduced in model 3, neither anemia nor SD‐ICH was not associated with a worse outcome. However, the association between the interaction term (anemia × SD‐ICH) and outcomes was even more potent (OR 4.33, 95% CI 1.60–11.9, *p *= 0.004), which was not observed in any other subtypes. Besides, the interactive effect analysis has demonstrated that no apparent detrimental effect of anemia over nonanemia was found across patients with different ages, genders, or different levels of consciousness or severity (all *p *> 0.2, Figure S2), suggesting that anemia was an independent factor for outcome in this cohort.

**TABLE 2:** Predictors of 3‐month death in ICH patients

	**Model 1**		**Model 2**		**Model 3**	
**Multivariable**	**OR (95% CI)**	***p***	**OR (95% CI)**	***p***	**OR (95% CI)**	***p***
Age (years)	1.02 (1.02–1.03)	0.000	1.03 (1.02–1.04)	<0.001	1.03 (1.02–1.04)	<0.001
Sex	0.96 (0.76–1.22)	0.739	0.93 (0.74–1.18)	0.561	0.93 (0.74–1.19)	0.575
GCS	0.75 (0.72–0.79)	<0.001	0.75 (0.72–0.79)	<0.001	0.75 (0.72–0.78)	<0.001
NIHSS	1.02 (1.00–1.04)	0.012	1.02 (1.01–1.04)	0.010	1.02 (1.01–1.04)	0.007
Hematoma volume (ml)	1.02 (1.01–1.03)	<0.001	1.02 (1.01–1.02)	<0.001	1.02 (1.01–1.02)	<0.001
Blood urea nitrogen	1.07 (1.04–1.11)	<0.001	1.06 (1.03–1.10)	<0.001	1.06 (1.02–1.09)	0.001
Comorbidities*	0.90 (0.65–1.24)	0.525	0.89 (0.62–1.26)	0.525	0.91 (0.63–1.29)	0.596
Intraventricular extension	1.82 (1.44–2.30)	<0.001	1.80 (1.42–2.28)	<0.001	1.84 (1.45–2.33)	<0.001
Surgical intervention	0.30 (0.22–0.39)	<0.001	0.29 (0.22–0.39)	<0.001	0.30 (0.22–0.39)	<0.001
Anemia	1.11 (0.86–1.43)	0.404	1.07 (0.83–1.38)	0.595	1.03 (0.68–1.55)	0.882
CAA			1.38 (0.94–2.02)	0.095	1.38 (0.86–2.18)	0.172
Structure lesion			1.43 (0.90–2.24)	0.127	1.74 (1.01–2.95)	0.041
Medication			1.93 (0.89–3.98)	0.084	1.60 (0.58–4.00)	0.341
Systemic disease			1.88 (1.13–3.12)	0.015	0.83 (0.38–1.74)	0.639
Undetermined			1.69 (1.28–2.23)	<0.001	1.77 (1.28–2.44)	<0.001
CAA × anemia					1.03 (0.47–2.25)	0.941
Structure lesion × anemia					0.55 (0.20–1.44)	0.230
Medication × anemia					1.55 (0.38–6.25)	0.540
Systemic disease × anemia					4.33 (1.60–11.9)	0.004
Undetermined × anemia					0.85 (0.46–1.59)	0.619

*Notes*: All multivariable models were adjusted for age, sex, Glasgow Coma Scale, National Institutes of Health Stroke Scale, hematoma volume and urea nitrogen, intraventricular extension and surgical interventions.

* Comorbidities were defined as any history of diseases such as coronary heart disease, congestive heart failure, cancer, leukocythemia, chronic pulmonary disease, diabetes mellitus, hepatic insufficiency or renal insufficiency. The structure lesion was structure lesion subtype, medication was medication subtype, and undetermined was the undetermined subtype according to the SMASH‐U etiology classification.

Abbreviations: CAA, cerebral amyloid angiopathy; CI, confidence interval; GCS, Glasgow Coma Scale; NIHSS, National Institutes of Health Stroke Scale; OR, odds ratio; *p*, *p* value.

### 2.4 Lower hemoglobin levels linearly correlate with worse outcomes in SD‐ICH

To investigate the effect of hemoglobin levels (or anemia) on clinical outcomes, patients of SD‐ICH were compared with those of hypertensive angiopathy (HA) subtype, the dominant etiological subtype of ICH. The patients of SD‐ICH were younger (56.1 vs. 60.9 years, *p *< 0.001), and presented with a higher frequency of anemia (56.8% vs. 24.1%, *p *< 0.001), lower hemoglobin level (12.2 vs. 13.7 g/dl, *p *< 0.001), and higher 3‐month death (36.1% vs. 14.7%; *p *< 0.001; Table 3 and Table S3) compared with those of HA. These basic characterizations were similar to that of the patients of consensus cluster 3. In multivariable models, the contribution of hemoglobin levels (or anemia) in 1‐month or 3‐month death was compared between systemic disease and HA subtypes (Tables S4–S6). It was demonstrated that hemoglobin levels (or anemia) were consistently associated with higher death in SD‐ICH (*p *= 0.009, *p *= 0.001, and *p *= 0.009, respectively) but not in HA (*p *= 0.417, *p *= 0.424, and *p *= 0.963, respectively).

**TABLE 3:** Baseline characteristics of patients of HA or systemic disease ICH subtypes

**Characteristics**	**HA *n* = 1,853**	**Systemic disease *n* = 236**	***p***
Age (years)	60.9 ± 12.6	56.1 ± 14.8	<0.001
Male, *n* (%)	1,157 (62.4%)	165 (69.9%)	0.029
Comorbidities and risk factors, *n* (%)			
HD history	127 (6.9%)	9 (3.8%)	0.100
Hypertension	1,853 (100.0%)	142 (60.2%)	<0.001
Hyperlipidemia	701 (45.9%)	74 (36.6%)	0.016
Diabetes mellitus	225 (12.1%)	26 (11.0%)	0.693
Chronic kidney disease	67 (3.6%)	107 (45.5%)	<0.001
Antithrombotics	45 (2.4%)	236 (100.0%)	<0.001
Alcohol	382 (20.6%)	40 (16.9%)	0.217
Smoking	460 (24.8%)	53 (22.5%)	0.474
Clinical factors			
GCS	13.0 (8.0, 15.0)	12.0 (6.0, 15.0)	<0.001
NIHSS	9.0 (4.0, 16.0)	11.0 (4.0, 24.0)	0.014
Hematoma volume (ml)	10.0 (4.5, 21.8)	16.0 (5.4, 33.4)	<0.001
SBP (mm Hg)	171.1 ± 26.8	170.0 ± 33.8	0.572
DBP (mm Hg)	99.6 ± 16.6	99.0 ± 18.7	0.559
Hemoglobin (g/dl)	13.7 ± 1.86	12.2 ± 2.79	<0.001
HCT	0.41 ± 0.05	0.37 ± 0.08	<0.001
Albumin (g/L)	41.5 ± 5.6	39.3 ± 6.3	<0.001
Platelet count (10^9^/L)	159 (121, 203)	108 (47, 162)	<0.001
PT, s	11.9 (11.1, 13.1)	12.4 (11.5, 13.8)	0.745
APTT, s	26.1 (23.2, 30.0)	27.5 (24.7, 31.5)	<0.001
Fibrinogen (g/L)	2.9 (2.3, 3.6)	3.0 (2.3, 3.9)	0.049
INR	1.02 (0.95, 1.09)	1.07 (1.00, 1.17)	<0.001
Blood glucose (mmol/L)	7.4 (6.2, 9.6)	7.3 (6.1, 9.6)	0.733
Creatinine (μmol/L)	70.8 (58.0, 86.0)	103.5 (72.0, 494.6)	<0.001
Hematoma location, *n* (%)			
BG or thalamus	1,526 (84.5%)	151(67.1%)	<0.001
Lobar	158 (8.9%)	70 (30.8%)	<0.001
Brainstem	176 (9.9%)	25 (11.0%)	0.680
Cerebellar	124 (7.0%)	10 (4.4%)	0.188
IVH	542 (29.2%)	97 (41.1%)	0.001
SAH	70 (3.8%)	20 (8.5%)	<0.001
Therapies and complications, *n*(%)			
Anti‐hypertension	1,266 (72.4%)	132 (60.6%)	<0.001
Dehydration	1,509 (86.3%)	190 (87.2%)	0.801
Surgical intervention	342 (18.5%)	26 (11.0%)	0.006
Respiratory infection	477 (25.7%)	53 (22.5%)	0.311

*Notes*: Descriptive statistics were calculated using mean ± SD or median (IQR) for continuous variables and frequencies for categorical variables. Abbreviations: APTT, activated partial thromboplastin time; DBP, diastolic blood pressure; GCS, Glasgow Coma Scale; HA, hypertensive angiopathy; HCT, hematocrit; HD, heart disease; INR, international normalized ratio; IQR, interquartile range; IVH, intraventricular hemorrhage; NIHSS, National Institutes of Health Stroke Scale; *p*, *p* value; PT, prothrombin time; SBP, systolic blood pressure; SD, standard deviation.

**FIGURE 3:**
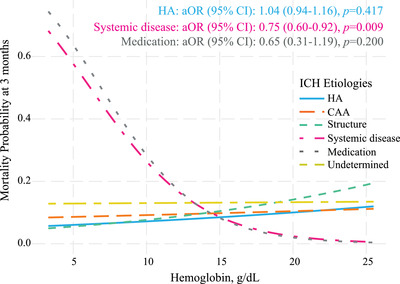
Predicted outcome probabilities by hemoglobin levels on admission in ICH patients with different etiological subtypes. Multivariable models were adjusted for age, sex, Glasgow Coma Scale, National Institutes of Health Stroke Scale, hematoma volume and urea nitrogen, intraventricular extension, and surgical interventions. Abbreviations: aOR, an adjusted odds ratio of outcome; 95% CI, 95% confidence interval; CAA, cerebral amyloid angiopathy; HA, hypertensive angiopathy.

**FIGURE 4:**
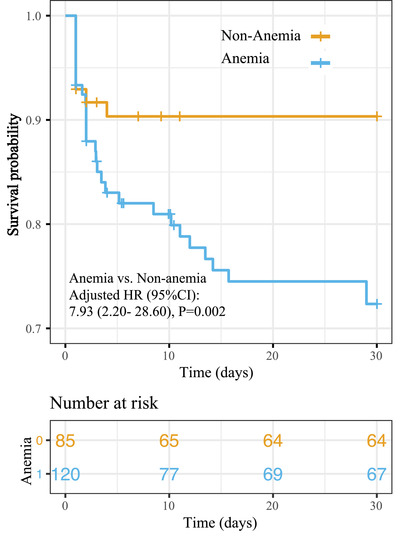
Cox proportional hazards regression curves for 1‐month survival rate according to anemia in ICH patients with systemic disease subtype. Hazard ratio (HR) was calculated using cox proportional hazard modeling with adjustment for potential confounders including age, sex, Glasgow Coma Scale, National Institutes of Health Stroke Scale, hematoma volume, and urea nitrogen, intraventricular extension and surgical interventions. Abbreviations: aOR, an adjusted odds ratio of outcome; 95% CI, 95% confidence interval.

We have further investigated the linear association between hemoglobin levels and clinical outcomes in different ICH subtypes. In SD‐ICH, a linear relationship between hemoglobin levels and 3‐month death was observed (hemoglobin levels per 1 g/dl: aOR 0.75, 95% CI 0.60–0.92; *p *= 0.009; Figure 3), which was not observed in other subtypes. Each 1 g/dl decrease of hemoglobin levels is calculated to be accompanied by 1.33‐folds increased risk of 3‐month death post‐ICH, which was comparable with 1.43‐fold for 1 score increase of the Glasgow Coma Scale (GCS) (aOR 0.70, 95% CI 0.54–0.86; *p *= 0.002, Table S4). Moreover, adjusted Cox regression indicated a decreased risk of 1‐month survival in the presence of anemia (adjusted hazard ratio [HR] 7.93, 95% CI 2.20–28.60, *p *= 0.002; Figure 4).

**FIGURE 5:**
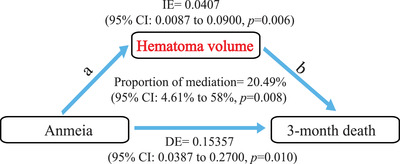
Mediation model of association of anemia with 3‐month death with hematoma volume on admission as a mediator in patients of systemic disease ICH subtype. The indirect effect (IE), or average causal mediation effects (ACME), was the effect of the exposure (anemia) on 3‐month death, mediated by hematoma volume on admission. The direct effect (DE), or average direct effects (ADE), was the remaining effect of the exposure on 3‐month death that was not mediated through hematoma volume. Path (a) was the effect of the anemia on hematoma volume. Path (b) was the effect of hematoma volume on 3‐month death.

Furthermore, when regressing anemia, hematoma volume, and outcome together, mediation analysis revealed that larger hematoma continued to be associated with poor outcomes after controlling for anemia (*p* = 0.004). The direct effect (DE) was 0.0407 (95%CI 0.0087–0.090, *p *= 0.006), and 20.49% of the mediation contributed to larger hematoma (*p *= 0.008; Figure 5 and Figure S3). This suggested that hematoma volume significantly mediated the effect of anemia with 3‐month death.

## 3 DISCUSSION

In this prospective cohort of 4643 consecutive ICH patients, anemia was not independently associated with worse clinical outcomes; in a subsequent analysis, anemia and lower hemoglobin levels were strongly correlated with worse outcomes in SD‐ICH. This relationship seemed to be mediated in part by hematoma volume on admission. Our findings suggested that hemoglobin levels might be an essential risk factor in SD‐ICH and therefore a modifiable factor in these patients.

Anemia and lower hemoglobin were found to be associated with a higher risk of worse outcomes (1‐ or 3‐month mortality) in ICH patients.^9^, ^15^, ^16^, ^17^ Although we found that the anemia group was associated with worse outcomes in the univariate analysis, the correlations were not significant after adjusting for other potential confounders such as age and hematoma volume. Moreover, moderate anemia (<11 g/dl) was not independently related to higher 3‐month death after adjusting confounders. The difference between our study and previous work might be due to the inclusion of additional confounders in the multivariate analysis, such as hematoma volume, GCS, intraventricular hemorrhage (IVH) or intraventricular extension, and surgical interventions, which were also critical determinants of prognosis in ICH.^6^, ^18^ Consistent with our results, one study of 2513 patients that did include GCS in the multivariate analysis also failed to find an independent association between anemia and 3‐month death.^16^ Similarly, Kumar et al. reported that anemia was associated with larger hematoma volume (*p* = 0.009) but not 1‐month death in ICH patients (aOR 1.5, 95% CI 0.9–2.4; *p* = 0.06; *N* = 685).^10^ Besides, Barlas et al. also found that, in male ICH patients, anemia was not significantly correlated with 3‐months death (aOR 1.39, 95% CI 0.81–2.39; *p* > 0.05; *N* = 513).^15^

The unique large set of ICH cohort here allows us to investigate the relationship between anemia and outcomes, taking the abnormal distribution of hemoglobin among ICH subtypes into consideration. The multiple etiologies of ICH patients might neutralize the potential effect of anemia in studies with small‐sample size. Here, we have employed the unsupervised consensus classification to investigate the association between anemia and ICH subtypes.^19^ We found that the consensus cluster 3 was mainly composed of SD‐ICH according to the SMASH‐U etiological classification, of which cluster the patients were characterized with lower hemoglobin levels, higher frequency of anemia, and higher death rate. Further multiple‐adjusted interaction analysis revealed that anemia interacted with ICH subtypes, suggesting that low hemoglobin levels might define SD‐ICH. In accordance with findings in the consensus cluster 3, the patients of SD‐ICH displayed similar characteristics; moreover, their worse outcomes were independently associated with anemia and low hemoglobin levels. Both 1‐ and 3‐month death of SD‐ICH were higher than those of HA subtype, consistent with previous studies.^7^, ^20^, ^21^, ^22^ Compared with HA, patients of SD‐ICH had younger age but presented worse general conditions, such as lower hemoglobin and albumin levels as well as higher urea nitrogen, which may be explained considering the higher frequency of comorbid illnesses in this subtype.

Lower hemoglobin levels were strongly and independently associated with worse outcomes in SD‐ICH. Our results indicated that hematoma volume might partially mediate the association between anemia and worse outcomes in SD‐ICH. Similarly, previous studies identified a positive relationship between anemia and larger baseline hematoma volumes in total ICH patients.^9^, ^10^ On the other side, instead of baseline hematoma volume, a recent study of 256 ICH patients has found that the associations of lower admission hemoglobin levels with increased hematoma expansion mediating worse outcomes in the subgroup analysis of 63 patients with hematoma expansion.^17^ The differential conclusion of these studies might result from different races, sample sizes, and study designs. Although hematoma volume might be more than a mediation factor, all these studies emphasized the possible mediation effect of hematoma, either baseline or expansion, on the association between lower hemoglobin levels and worse outcomes post‐ICH.

It is plausible that the pathophysiologic basis for the association between either baseline hematoma volume or hematoma expansion and the lower hemoglobin levels may be related to previously shown evidence of coagulopathy and prolonged bleeding in anemic patients.^23^ In our study, the patients of SD‐ICH also demonstrated abnormal coagulation. The strong effect of lower hemoglobin levels in SD‐ICH might be due to the high prevalence of comorbid illness and the resulting worse general condition, which makes the patients of SD‐ICH, compared with those of other subtypes, more sensitive to the impaired function of reduced hemoglobin levels, such as neuronal tissue hypoxia, metabolic distress, and cell energy dysfunction.^24^, ^25^, ^26^ Our results indicated that lower hemoglobin levels were linearly correlated with worse outcomes in SD‐ICH, implicating that hemoglobin‐raising strategies such as an iron supplement or blood transfusion might be beneficial for specific ICH subgroup patients.^27^

The main strength of our study is that it includes a large, heterogeneous patient population who underwent rigorous, systemic evaluations early after the onset of acute ICH. At the same time, our work presents some limitations. First, we included only tertiary hospitals in our study, leading to the loss of patients with less severity. Second, the proportion of patients with medication‐related ICH was relatively small. In previous studies of Asian patients with atrial fibrillation, only about 20% had taken anticoagulants, and relatively few showed an international normalized ratio (INR) in the therapeutic range.^28^, ^29^, ^30^ Previous studies have already pointed out a lower incidence of medication‐related ICH in Asian populations (2–3%) than in Caucasian populations (14%).^30^, ^31^, ^32^ This may be related to the lower use of anticoagulation and thrombolysis in Asian clinics. Third, anemia was diagnosed based on a single measurement on admission, which occurred at different times since symptom onset among different patients. This increases the risk that our results are affected by regression attenuation bias.

In summary, in this large multicenter ICH cohort, our study revealed an etiology‐specific association between low hemoglobin levels on admission and poor 3‐month outcomes. Our data suggested that low hemoglobin on admission is strongly associated with worse outcomes in SD‐ICH, which might be mediated by the larger hematoma volume on admission. Further studies are needed to replicate our results and investigate whether the intervention of hemoglobin levels, either by blood transfusion or iron supplement, would benefit the patients of SD‐ICH.

## 4 METHODS

### 4.1 Study design and participants

We used a cohort study design based on a prospective, multicenter, hospital‐based registry that collected data on patients with acute ICH admitted to 21 tertiary hospitals across a wide range of cities in China from January 2012 to December 2015. A total of 4904 patients who received a clinical diagnosis of spontaneous ICH confirmed by brain imaging were screened. The study was approved by the Biomedical Research Ethics Committee and the Committee on Human Research of West China Hospital, Sichuan University (2013 [124]).^33^ Informed consent was obtained from participants or their guardians.

Patients with first‐ever ICH were consecutively recruited. Patients were included if they (1) were at least 18 years old, (2) had been diagnosed with ICH based on noncontrast computed tomography (NCCT) performed within 72 h from the presumed symptom onset. The patients with aneurysmal subarachnoid hemorrhages and lobar bleeding were included. Patients were excluded (1) if they were diagnosed with traumatic ICH, primary subdural/epidural hematoma, intracranial venous thrombosis, or hemorrhage due to a tumor or recurrent ICH; (2) if they were diagnosed with hemorrhagic transformation of a cerebral infarction; or (3) hemoglobin measurements on admission were missing.

### 4.2 Demographic and clinical data collection

Information about baseline demographic characteristics was obtained predominantly through in‐person interviews. In‐hospital details, including clinical features and diagnosis, were obtained through medical records and interviews with patients or their families. Follow‐up details were obtained primarily through telephone interviews at one and three months after the stroke. Medical history variables included the existence of any of the following: hypertension, diabetes, hyperlipidemia, stroke, heart disease (including any history of atrial fibrillation/heart attack/myocardial infarction, angina, coronary heart disease, or valvular heart disease), either self‐reported or diagnosed in‐hospital before ICH onset. Comorbidities were defined if the patient had coronary heart disease, congestive heart failure, cancer, leukocythemia, chronic pulmonary disease, diabetes mellitus, hepatic insufficiency, or renal insufficiency. Surgical interventions were indicated according to the Guidelines for the management of spontaneous ICH.^34^ Brain CT scans were performed in all patients on admission. Hematoma volume was determined by the formula of ellipsoids (A*B*C/2).^35^ Hemoglobin levels were measured on admission using the hemiglobincyanide method. According to the World Health Organization (WHO) criteria, anemia was defined as a hemoglobin concentration below 12 g/dl in females and below 13 g/dl in males; whereas moderate anemia was defined as a hemoglobin concentration below 11 g/dl in females and below 12 g/dl in males. The primary outcome in these analyses was death at three months. The follow‐up period was three months, and the follow‐up rate was 93% (4307/4643).

Etiological subtypes of ICH were classified according to SMASH‐U criteria (structural lesion, medication, amyloid angiopathy, systemic/other diseases, hypertension, undetermined).^7^ The SD‐ICH subtype was defined by the presence of thrombocytopenia or liver cirrhosis as previously described,^7^ or by the presence of nondrug‐induced coagulopathy or renal failure (stage 5 chronic kidney disease or kidney disease requiring dialysis), both of which are risk factors for spontaneous ICH.^20^, ^36^ HA subtype was defined as deep or infratentorial hemorrhage with pre‐ICH hypertension history. Hypertension was defined as any recorded hypertension diagnosis or pre‐existing BP ≥140/90 mm Hg on at least three measurements at rest on at least two separate health care visits for more than one month before ICH onset, either on or off antihypertensive therapy. Two experts classified all cases independently. The inter‐evaluator agreement for ICH etiologic classification was high (*κ* = 0.93).

### 4.3 Statistical analysis

Categorical variables were presented as counts (%), and the continuous or discrete variables were presented as mean (standard deviation, SD) or median (interquartile range, IQR). Student's *t‐*test, the *χ* ^2^ test, ANOVA, Mann–Whitney *U*‐test, Fisher's exact test, and Kruskal–Wallis test were used for univariate analysis among groups with relevant variables as appropriate. Associations of clinical characteristics with death were analyzed using logistic regression models, whereas associations of clinical characteristics with mRS were analyzed using ordinal logistic regression. Data are reported as ORs and 95% CI. Consensus cluster is an unsupervised clustering method for cluster discovery and in conjunction with resampling techniques.^37^ The consensus across multiple runs of a clustering algorithm for a consensus cluster depends on the separation of clusters in the matrix and the average silhouette width between different clusters. The cumulative distribution function (CDF) of the consensus matrix is the indicator to assess the stability of the discovered clusters. Based on the analysis, we can discriminate against the individuals. The goal of the analysis is to divide the cohort into separated sub‐cohorts with their features and to prove the specific effect of hemoglobin on the ICH subtype. HR was calculated using cox proportional hazard modeling with adjustment for age, sex, GCS, National Institutes of Health Stroke Scale, hematoma volume and urea nitrogen, intraventricular extension, and surgical interventions.

Mediation analysis was performed to estimate whether hematoma volume (as the mediator) was the mediator for any relationship between the hemoglobin level (independent variable) and poor outcome (dependent variable) by analyzing all three variables together with four steps: (1) path c: the total effect of the elevated hemoglobin level (group factor) on the outcome; (2) path a: the group effect on hematoma volume; (3) path b: the correlation between hematoma volume and outcomes, after controlling for the group factor; and (4) the *a* × *b* effect, which was referred to as the indirect effect (IE) and was indicative of whether the predictor–outcome relationship was significantly reduced after controlling for the mediator. If all four tests reached the level of significance, hematoma volume was considered to significantly mediate the group effect on the poor outcome. The *a* × *b* IE was evaluated using the R mediation package with 5,000 repetitions that were used to estimate the CI for the IEs. An empirical 95% CI that did not include zero indicated significance at the 0.05 level. Analyses were performed using the R mediation package. To systematically identify potential confounders in the multivariable model, we selected variables using a “least absolute shrinkage and selection operator” regularizer, combined with univariable analysis and clinical significance. The least absolute shrinkage and selection operator calculation was conducted using the glmnet algorithm in R, where appropriate, aORs were reported. Age, GCS, NIHSS, hematoma volume, urea nitrogen, plus intraventricular hemorrhage (IVH) or intraventricular extension , and surgical interventions were identified as confounding variables in the total cohort, which were adjusted in all multivariable models. Two‐sided *p* values are reported, with *p* < 0.05 considered statistically significant in all tests unless another threshold was given. All statistical analyses were performed in R Core Team (2017) (R: A language and environment for statistical computing. R Foundation for Statistical Computing, Vienna, Austria; https://www.R‐project.org/.).

## CONFLICT OF INTEREST

The authors declare that they have no conflicts of interest.

## ETHICS APPROVAL

The study was approved by the Biomedical Research Ethics Committee and the Committee on Human Research of West China Hospital, Sichuan University (Reference No. 2013 [124]). Informed consent was obtained from participants or their guardians.

## AUTHOR CONTRIBUTIONS

ML, SZ, and PL designed and conducted the cohort study; SZ, HC, XL, YC, BW collected the data and constructed the database; SZ, YS and YL analyzed the data; SZ, ML, and PL wrote the paper. All authors reviewed the manuscript.

## Supporting information

Supporting information

## Data Availability

The data that support the findings of this study are available from the corresponding author upon reasonable request.
