# Supplementary material for: Low hemoglobin is associated with worse outcomes via larger hematoma volume in intracerebral hemorrhage due to systemic disease
Source: MedComm (2020). 2022 Feb 23;3(1):e96. doi: 10.1002/mco2.96 (PMC8906467; doi:10.1002/mco2.96)
Supplement: Supplementary file 1 — Supporting information [file MCO2-3-e96-s001.docx]

***Supplementary materials:***

**Low hemoglobin is associated with worse outcomes via larger hematoma volume in intracerebral hemorrhage due to systemic disease.**

Shuting Zhang, MD, PhD ^1, #^, Yang Shu, PhD ^2, #^, Yunlong Chen, BM ^3^, Xiaoyang Liu, BM ^3^, Yu Liu, M.S. ^2^, Yajun Cheng, MD ^1^, Bo Wu, MD, PhD ^1^, Peng Lei, PhD ^1, 2, *^ and Ming Liu, MD, PhD ^1, *^

**Supplementary table numbers:** 6

**Supplementary figure numbers:** 3

**Table S1. Multivariate analysis of the association of anemia or moderate anemia with outcomes in ICH patients.**

|  | **Multivariable** | **1-month death** | |  | **3-month death** | |  |
| --- | --- | --- | --- | --- | --- | --- | --- |
|  |  | **OR (95% CI)** | ***p*** |  | **OR (95% CI)** | ***p*** |  |
| **Total** | Anemia | 1·15 (0·85-1·57) | 0·361 |  | 1.11 (0.86-1.43) | 0.404 |  |
|  | Moderate anemia | 1·35 (0·93-1·95) | 0·112 |  | 1·33 (0·97-1·82) | 0·070 |  |
|  |  |  |  |  |  |  |  |
| **Female** | Anemia | 1·19 (0·71-1·97) | 0·508 |  | 1·19 (0·80-1·77) | 0·377 |  |
|  | Moderate anemia | 1·02 (0·52-1·92) | 0·958 |  | 1·31 (0·77-2·19) | 0·310 |  |
|  |  |  |  |  |  |  |  |
| **Male** | Anemia | 1·21 (0·81-1·78) | 0·340 |  | 1·12 (0·82-1·53) | 0·477 |  |
|  | Moderate anemia | 1·65 (1·04-2·59) | 0·031 |  | 1·38 (0·93-2·03) | 0·106 |  |

All multivariable models were adjusted for age, sex, Glasgow Coma Scale, National Institutes of Health Stroke Scale, hematoma volume and urea nitrogen, intraventricular extension and surgical interventions. Abbreviations: OR, odds ratio; CI, confidence interval; *p*, *p* value.

**Table S2. Baseline characteristics of ICH patients of three consensus clusters.**

| **Characteristics** | **Cluster 1 (N = 1,715)** | **Cluster 2 (N = 2,860)** | **Cluster 3 (N = 68)** | ***p*** |
| --- | --- | --- | --- | --- |
| Age, years | 54.9 ± 15.7 | 60.5 ± 14.6 | 52.1 ± 12.1 | <0.001 |
| Male, n (%) | 1,030 (60.1%) | 1,891 (66.1%) | 51 (75.0%) | <0.001 |
| **Comorbidities and risk factors, n (%)** | |  |  |  |
| HD History | 136 (7.9%) | 182 (6.4%) | 1 (1.5%) | 0.026 |
| Hypertension | 951 (55.5%) | 1,614 (56.4%) | 54 (79.4%) | <0.001 |
| Hyperlipidemia | 639 (43.8%) | 853 (36.1%) | 20 (37.7%) | <0.001 |
| Diabetes mellitus | 142 (8.3%) | 240 (8.4%) | 10 (14.7%) | 0.172 |
| Chronic kidney disease | 52 (3.0%) | 116 (4.1%) | 68 (100.0%) | <0.001 |
| Anti-thrombotics | 124 (7.2%) | 219 (7.7%) | 63 (92.6%) | <0.001 |
| Alcohol | 329 (19.2%) | 581 (20.3%) | 8 (11.8%) | 0.160 |
| Smoking | 399 (23.3%) | 745 (26.0%) | 16 (23.5%) | 0.104 |
| **Clinical status** |  |  |  |  |
| GCS | 14.0 (8.0, 15.0) | 13.0 (8.0, 15.0) | 14.0 (6.0, 15.0) | 0.031 |
| NIHSS | 8.0 (2.0, 16.0) | 9.0 (3.0, 17.0) | 9.0 (4.0, 22.0) | 0.076 |
| Hematoma volume, mL | 12.0 (5.0, 26.1) | 13.2 (5.2, 27.5) | 18.8 (5.4, 35.3) | 0.009 |
| SBP, mmHg | 161.0 ± 31.6 | 162.5 ± 31.1 | 185.1 ± 31.0 | <0.001 |
| DBP, mmHg | 95.8 ± 18.5 | 94.2 ± 17.7 | 105.1 ± 19.2 | <0.001 |
| Hemoglobin, g/dL | 13.8 ± 2.0 | 13.4 ± 2.0 | 10.7 ± 2.0 | <0.001 |
| HCT | 0.41 ± 0.06 | 0.40 ± 0.06 | 0.33 ± 0.06 | <0.001 |
| Albumin, g/L | 41.6 ± 5.5 | 41.4 ± 5.4 | 39.7 ± 5.3 | 0.015 |
| Platelet count, 10^9^/L | 220.0 (197.0, 255.5) | 128.0 (101.0, 154.0) | 129.0 (107.5, 154.2) | <0.001 |
| PT, s | 11.9 (11.1, 13.0) | 11.9 (11.2, 13.0) | 1 (11.6, 12.9) | 0.455 |
| APTT, s | 26.2 (23.0, 30.2) | 26.0 (23.3, 29.5) | 7 (24.5, 29.9) | 0.543 |
| Fibrinogen, g/L | 3.0 (2.4, 3.7) | 2.8 (2.3, 3.5) | 3.1 (2.6, 4.0) | <0.001 |
| INR | 1.02 (0.96, 1.10) | 1.04 (0.97, 1.11) | 1.06 (1.00, 1.11) | 0.278 |
| Blood glucose, mmol/L | 7.3 (6.1, 9.3) | 7.3 (6.1, 9.4) | (5.7, 9.9) | 0.587 |
| Creatinine, umol/L | 68.3 (55.8, 83.0) | 71.7 (59.0, 87.0) | 841.0 (577.0, 1,005.5) | <0.001 |
| **Hematoma Location, n (%)** |  |  |  |  |
| BG or thalamus | 969 (61.8%) | 1,707 (64.2%) | 43 (66.2%) | 0.483 |
| Lobar | 553 (33.4%) | 913 (33.3%) | 22 (33.8%) | 0.994 |
| Brainstem | 146 (8.8%) | 218 (8.0%) | 9 (13.8%) | 0.165 |
| Cerebellar | 112 (6.8%) | 163 (5.9%) | 0 (0.0%) | 0.063 |
| IVH | 523 (30.5%) | 994 (34.8%) | 23 (33.8%) | 0.166 |
| SAH | 124 (7.2%) | 234 (8.2%) | 2 (2.9%) | 0.012 |
| **Therapies and complications, n (%)** | |  |  |  |
| Anti-hypertension | 961 (58.9%) | 1,569 (58.6%) | 48 (77.4%) | 0.011 |
| Dehydration | 1,434 (87.9%) | 2,396 (89.4%) | 49 (79.0%) | 0.016 |
| Surgical intervention | 472 (27.5%) | 744 (26.0%) | 2 (2.9%) | <0.001 |
| Respiratory infection | 379 (22.1%) | 616 (21.5%) | 12 (17.6%) | 0.649 |

Descriptive statistics were calculated using mean ± SD or median (IQR) for continuous variables and frequencies for categorical variables. Abbreviations: HD, heart disease; GCS, Glasgow Coma Scale; NIHSS, National Institutes of Health Stroke Scale; SBP, systolic blood pressure; DBP, diastolic blood pressure; HCT, hematocrit; INR, International normalized ratio; HA, hypertensive angiopathy; SD, standard deviation; IQR, interquartile range; *p, p* value.

**Table S3. Baseline characteristics of study population, according to etiological ICH subtypes.**

HA, hypertensive angiopathy; CAA, cerebral amyloid angiopathy; GCS, Glasgow Coma Scale; NIHSS, National Institutes of Health Stroke Scale; SBP, systolic blood pressure; DBP, diastolic blood pressure; HD, heart disease; HCT, hematocrit; PT, prothrombin time; APTT, activated partial thromboplastin time; INR, International normalized ratio; BG, basal ganglia; SAH, subarachnoid hemorrhage; IVH, intraventricular hemorrhage.

| **Characteristics** | **HA** | **Systemic disease** | **CAA** | **Structural lesion** | **Medication** | **Undetermined** | ***p*** |
| --- | --- | --- | --- | --- | --- | --- | --- |
|  | **n=1,853** | **n=236** | **n= 528** | **n = 631** | **n = 110** | **n = 1285** |  |
| Age, years | 60.9 ± 12.6 | 56.1 ± 14.8 | 69.2 ± 9.3 | 45.1 ± 16.4 | 64.1 ± 13.5 | 56.4 ± 15.2 | < 0.0001 |
| Male, n (%) | 1,157 (62.4%) | 165 (69.9%) | 357 (67.6%) | 354 (56.1%) | 73 (66.4%) | 866 (67.4%) | < 0.0001 |
| **Comorbidities and risk factors, n (%)** | |  |  |  |  |  |  |
| HD History | 127 (6.9%) | 9 (3.8%) | 42 (8.0%) | 7 (1.1%) | 100 (90.9%) | 34 (2.6%) | < 0.0001 |
| Hypertension | 1,853 (100.0%) | 142 (60.2%) | 286 (54.2%) | 106 (16.8%) | 77 (70.0%) | 155 (12.1%) | < 0.0001 |
| Hyperlipidemia | 701 (45.9%) | 74 (36.6%) | 10 (1.9%) | 2 (0.3%) | 24 (21.8%) | 9 (0.7%) | < 0.0001 |
| Diabetes mellitus | 225 (12.1%) | 26 (11.0%) | 47 (8.9%) | 12 (1.9%) | 34 (30.9%) | 48 (3.7%) | < 0.0001 |
| Chronic kidney disease | 67 (3.6%) | 107(45.5%) | 13(2.5%) | 6(1.0%) | 5(4.6%) | 38 (3.0%) | < 0.0001 |
| Antithrombotics | 45 (2.4%) | 236 (100.0%) | 4 (0.8%) | 0 (0.0%) | 110 (100.0%) | 0 (0.0%) | < 0.0001 |
| Alcohol | 382 (20.6%) | 40 (16.9%) | 82 (15.5%) | 89 (14.1%) | 49 (44.5%) | 276 (21.5%) | < 0.0001 |
| Smoking | 460 (24.8%) | 53 (22.5%) | 118 (22.3%) | 149 (23.6%) | 46 (41.8%) | 334 (26.0%) | 0.0009 |
| **Clinical factors** |  |  |  |  |  |  |  |
| GCS | 13.0 (8.0, 15.0) | 12.0 (6.0, 15.0) | 13.0 (9.0, 15.0) | 14.0 (11.0, 15.0) | 12.0 (11.0, 14.0) | 13.0 (7.0, 15.0) | < 0.0001 |
| NIHSS | 9.0 (4.0, 16.0) | 11.0 (4.0, 24.0) | 6.0 (2.0, 13.0) | 3.0 (0.0, 11.0) | 12.0 (8.0, 15.8) | 10.0 (3.0, 21.0) | < 0.0001 |
| Hematoma volume, mL | 10.0 (4.5, 21.8) | 16.0 (5.4, 33.4) | 21.0 (9.4, 35.1) | 11.9 (4.9, 23.9) | 2.5 (8.0, 27.9) | 14.3 (5.4, 30.2) | < 0.0001 |
| SBP, mmHg | 171.1 ± 26.8 | 170.0 ± 33.8 | 159.6 ± 27.9 | 135.6 ± 26.5 | 171.6 ± 29.5 | 161.6 ± 33.1 | < 0.0001 |
| DBP, mmHg | 99.6 ± 16.6 | 99.0 ± 18.7 | 90.6 ± 15.6 | 82.2 ± 15.4 | 95.8 ± 16.0 | 95.5 ± 19.0 | < 0.0001 |
| Hemoglobin, g/dL | 13.7 ± 1.86 | 12.2 ± 2.79 | 13.3 ± 1.9 | 13.3 ± 1.8 | 12.9 ± 1.7 | 13.8 ± 2.1 | < 0.0001 |
| HCT | 0.41 ± 0.05 | 0.37 ± 0.08 | 0.40 ± 0.06 | 0.40 ± 0.05 | 0.38 ± 0.05 | 0.41 ± 0.06 | < 0.0001 |
| Albumin, g/L | 41.5 ± 5.6 | 39.3 ± 6.3 | 40.8 ± 5.1 | 42.0 ± 4.7 | 38.4 ± 4.7 | 42.1 ± 5.4 | < 0.0001 |
| Platelet count, 10^9^/L | 159 (121, 203) | 108 (47, 162) | 152 (112, 191) | 169(129, 217) | 189 (127, 215) | 159 (121, 206) | < 0.0001 |
| PT, s | 11.9 (11.1, 13.1) | 12.4 (11.5, 13.8) | 11.9 (11.1, 12.8) | 11.9 (11.3, 12.6) | 14.3 (12.5, 20.0) | 11.8 (11.1, 12.7) | < 0.0001 |
| APTT, s | 26.1 (23.2, 30.0) | 27.5 (24.7, 31.5) | 25.8 (23.0, 29.4) | 26.2 (23.6, 29.4) | 27.6 (24.0, 32.4) | 25.7 (22.9, 29.3) | < 0.0001 |
| Fibrinogen, g/L | 2.9 (2.3, 3.6) | 3.0 (2.3, 3.9) | 3.0 (2.5, 3.8) | 2.8 (2.2, 3.5) | 2.7 (2.4, 3.0) | 2.9 (2.3, 3.5) | < 0.0001 |
| INR | 1.02 (0.95, 1.09) | 1.07 (1.00, 1.17) | 1.03 (0.97, 1.10) | 1.05 (0.99, 1.11) | 2.40 (2.13, 2.55) | 1.02 (0.96, 1.09) | < 0.0001 |
| Blood glucose, mmol/L | 7.4 (6.2, 9.6) | 7 (6.1, 9.6) | 7.43 (6.27, 9.58) | 6.93 (5.84, 8.29) | 8.40 (7.40, 9.40) | 7.32 (5.99, 9.46) | < 0.0001 |
| Creatinine, umol/L | 70.8 (58.0, 86.0) | 103.5 (72.0, 494.6) | 71.8 (60.2, 87.0) | 65.0 (53.5, 79.0) | 79.5 (70.3, 101.3) | 70.2 (58.3, 84.0) | < 0.0001 |
| **Hematoma Location, n (%)** | |  |  |  |  |  |  |
| BG or thalamus | 1,526 (84.5%) | 151(67.1%) | 39(8.5%) | 116(24.0%) | 72(71.3%) | 815 (67.1%) | < 0.0001 |
| Lobar | 158 (8.9%) | 70 (30.8%) | 523 (100.0%) | 385 (63.1%) | 26 (24.5%) | 326 (26.8%) | < 0.0001 |
| Brainstem | 176 (9.9%) | 25 (11.0%) | 3(0.6%) | 34(5.6%) | 6(5.7%) | 129 (10.6%) | < 0.0001 |
| Cerebellar | 124 (7.0%) | 10 (4.4%) | 33(6.3%) | 40(6.6%) | 6(5.7%) | 62 (5.1%) | 0.3212 |
| IVH | 542 (29.2%) | 97 (41.1%) | 122 (23.1%) | 262 (41.5%) | 32 (29.1%) | 485 (37.7%) | < 0.0001 |
| SAH | 70 (3.8%) | 20 (8.5%) | 38 (7.2%) | 144 (22.8%) | 4 (3.6%) | 84 (6.5%) | < 0.0001 |
| **Therapies and complications, n(%)** | |  |  |  |  |  |  |
| Anti-hypertension | 1,266 (72.4%) | 132 (60.6%) | 289 (57.9%) | 189 (31.6%) | 58 (55.8%) | 644 (53.5%) | < 0.0001 |
| Dehydration | 1,509 (86.3%) | 190 (87.2%) | 455 (91.2%) | 547 (91.3%) | 72 (69.2%) | 1,106 (91.9%) | < 0.0001 |
| Surgical intervention | 342 (18.5%) | 26 (11.0%) | 109 (20.6%) | 428 (67.8%) | 21 (19.1%) | 292 (22.7%) | < 0.0001 |
| Respiratory infection | 477 (25.7%) | 53 (22.5%) | 114 (21.6%) | 79 (12.5%) | 33 (30.0%) | 251 (19.5%) | < 0.0001 |

**Table S4. Multivariate analysis of association between hemoglobin levels and 3-month death in patients of Hypertensive angiopathy (HA) or systemic disease ICH subtypes.**

| **3-month Death** | **Hypertensive angiopathy** | | | |  | **Systemic disease** | | | |
| --- | --- | --- | --- | --- | --- | --- | --- | --- | --- |
|  | **b** | **SE** | **OR (95%CI)** | ***p*** |  | **b** | **SE** | **OR (95%CI)** | ***p*** |
| Age, years | 0.036 | 0.008 | 1.04 (1.02-1.05) | 0.000 |  | 0.008 | 0.018 | 1.01 (0.97-1.05) | 0.656 |
| Sex | -0.041 | 0.202 | 0.96 (0.65-1.43) | 0.841 |  | -1.041 | 0.514 | 0.35 (0.12-0.95) | 0.043 |
| GCS | -0.284 | 0.037 | 0.75 (0.70-0.81) | 0.000 |  | **-0.358** | **0.118** | **0.70 (0.54-0.86)** | **0.002** |
| NIHSS | 0.039 | 0.013 | 1.04 (1.01-1.07) | 0.004 |  | -0.034 | 0.049 | 0.97 (0.87-1.06) | 0.497 |
| Hematoma volume, mL | 0.017 | 0.005 | 1.02 (1.01-1.03) | 0.001 |  | 0.039 | 0.014 | 1.04 (1.01-1.07) | 0.005 |
| Blood urea nitrogen | 0.070 | 0.032 | 1.07 (1.01-1.14) | 0.030 |  | 0.015 | 0.029 | 1.02 (0.96-1.07) | 0.591 |
| Comorbidities* | -0.305 | 0.268 | 0.74 (0.43-1.23) | 0.256 |  | 0.940 | 0.609 | 2.56 (0.77-8.60) | 0.123 |
| Intraventricular extension | 0.424 | 0.199 | 1.53 (1.03-2.25) | 0.033 |  | 0.844 | 0.496 | 2.33 (0.88-6.25) | 0.089 |
| Surgical intervention | -1.068 | 0.238 | 0.34 (0.21-0.54) | 0.000 |  | -1.956 | 0.772 | 0.14 (0.03-0.58) | 0.011 |
| **Hemoglobin, g/dL** | **0.043** | **0.053** | **1.04 (0.94-1.16)** | **0.417** |  | **-0.284** | **0.108** | **0.75 (0.60-0.92)** | **0.009** |

Multivariable models were adjusted for age, sex, Glasgow Coma Scale, National Institutes of Health Stroke Scale, hematoma volume and urea nitrogen, intraventricular extension and surgical interventions. Comorbidities* were defined as any history of diseases like coronary heart disease, congestive heart failure, cancer, leukocythemia, chronic pulmonary disease, diabetes mellitus, hepatic insufficiency or renal insufficiency. Abbreviations: OR, odds ratio; CI, confidence interval; *p, p* value; GCS, Glasgow Coma Scale; NIHSS, National Institutes of Health Stroke Scale.

**Table S5. Multivariate analysis of association between hemoglobin levels and 1-month death in patients of HA or systemic disease ICH subtypes.**

| **1-month Death** | **Hypertensive angiopathy** | | | |  | **Systemic disease** | | | |
| --- | --- | --- | --- | --- | --- | --- | --- | --- | --- |
|  | **b** | **SE** | **OR (95%CI)** | ***p*** |  | **b** | **SE** | **OR (95%CI)** | ***p*** |
| Age | 0.043 | 0.011 | 1.04 (1.02-1.07) | 0.000 |  | 0.022 | 0.018 | 1.02 (0.99-1.06) | 0.221 |
| Sex | -0.070 | 0.266 | 0.93 (0.55-1.58) | 0.792 |  | 0.084 | 0.521 | 1.09 (0.40-3.13) | 0.871 |
| GCS | -0.169 | 0.051 | 0.84 (0.76-0.93) | 0.001 |  | -0.031 | 0.096 | 0.97 (0.80-1.18) | 0.749 |
| NIHSS | 0.074 | 0.017 | 1.08 (1.04-1.11) | 0.000 |  | 0.071 | 0.045 | 1.07 (0.99-1.18) | 0.117 |
| Hematoma | 0.007 | 0.005 | 1.01 (1.00-1.02) | 0.198 |  | -0.011 | 0.012 | 0.99 (0.97-1.01) | 0.346 |
| Blood urea nitrogen | 0.062 | 0.038 | 1.06 (0.99-1.14) | 0.098 |  | -0.007 | 0.028 | 0.99 (0.94-1.05) | 0.815 |
| Comorbidities* | 0.214 | 0.325 | 1.24 (0.64-2.30) | 0.510 |  | 0.094 | 0.620 | 1.10 (0.31-3.60) | 0.879 |
| Intraventricular extension | 0.815 | 0.259 | 2.26 (1.36-3.77) | 0.002 |  | 1.346 | 0.554 | 3.84 (1.34-11.9) | 0.015 |
| Surgical intervention | -0.555 | 0.301 | 0.57 (0.31-1.02) | 0.065 |  | -1.767 | 1.134 | 0.17 (0.01-1.10) | 0.119 |
| **Hemoglobin, g/dL** | **0.056** | **0.070** | **1.06 (0.92-1.22)** | **0.424** |  | **-0.388** | **0.119** | **0.68 (0.53-0.84)** | **0.001** |

Multivariable models were adjusted for age, sex, Glasgow Coma Scale, National Institutes of Health Stroke Scale, hematoma volume and urea nitrogen, intraventricular extension and surgical interventions. Comorbidities* were defined as any history of heart disease like coronary artery disease or atrial fibrillation, diabetes mellitus. Abbreviations: OR, odds ratio; CI, confidence interval; *p, p* value; GCS, Glasgow Coma Scale; NIHSS, National Institutes of Health Stroke Scale.

**Table S6. Multivariate analysis of association between anemia and 3-month death in patients of HA or systemic disease ICH subtypes.**

| **3-month Death** | **Hypertensive angiopathy** | | | |  | **Systemic disease** | | | |
| --- | --- | --- | --- | --- | --- | --- | --- | --- | --- |
|  | **b** | **SE** | **OR (95%CI)** | ***p*** |  | **b** | **SE** | **OR (95%CI)** | ***p*** |
| Age, years | 0.035 | 0.008 | 1.04 (1.02-1.05) | 0.000 |  | 0.005 | 0.019 | 1.00 (0.97-1.04) | 0.789 |
| Sex | 0.013 | 0.193 | 1.01 (0.70-1.48) | 0.948 |  | -1.178 | 0.508 | 0.31 (0.11-0.82) | 0.021 |
| GCS | -0.285 | 0.037 | 0.75 (0.70-0.81) | 0.000 |  | -0.361 | 0.114 | 0.70 (0.54-0.86) | 0.002 |
| NIHSS | 0.038 | 0.013 | 1.04 (1.01-1.07) | 0.004 |  | -0.035 | 0.047 | 0.97 (0.87-1.06) | 0.465 |
| Hematoma volume, mL | 0.017 | 0.005 | 1.02 (1.01-1.03) | 0.002 |  | 0.042 | 0.014 | 1.04 (1.02-1.07) | 0.003 |
| Blood urea nitrogen | 0.068 | 0.032 | 1.07 (1.00-1.14) | 0.035 |  | 0.025 | 0.027 | 1.03 (0.97-1.08) | 0.356 |
| Comorbidities* | -0.320 | 0.269 | 0.73 (0.42-1.21) | 0.234 |  | 1.066 | 0.618 | 2.90 (0.86-9.95) | 0.084 |
| Intraventricular extension | 0.437 | 0.199 | 1.55 (1.05-2.28) | 0.028 |  | 0.753 | 0.484 | 2.12 (0.82-5.55) | 0.119 |
| Surgical intervention | -1.076 | 0.238 | 0.34 (0.21-0.54) | 0.000 |  | -1.865 | 0.770 | 0.15 (0.03-0.64) | 0.015 |
| **Anemia** | **0.010** | **0.219** | **1.01 (0.65-1.54)** | **0.963** |  | **1.398** | **0.532** | **4.05 (1.49-12.2)** | **0.009** |

Multivariable models were adjusted for age, sex, Glasgow Coma Scale, National Institutes of Health Stroke Scale, hematoma volume and urea nitrogen, intraventricular extension and surgical interventions. Comorbidities* were defined as any history of diseases like coronary heart disease, congestive heart failure, cancer, leukocythemia, chronic pulmonary disease, diabetes mellitus, hepatic insufficiency or renal insufficiency. Abbreviations: OR, odds ratio; CI, confidence interval; *p, p* value; GCS, Glasgow Coma Scale; NIHSS, National Institutes of Health Stroke Scale.


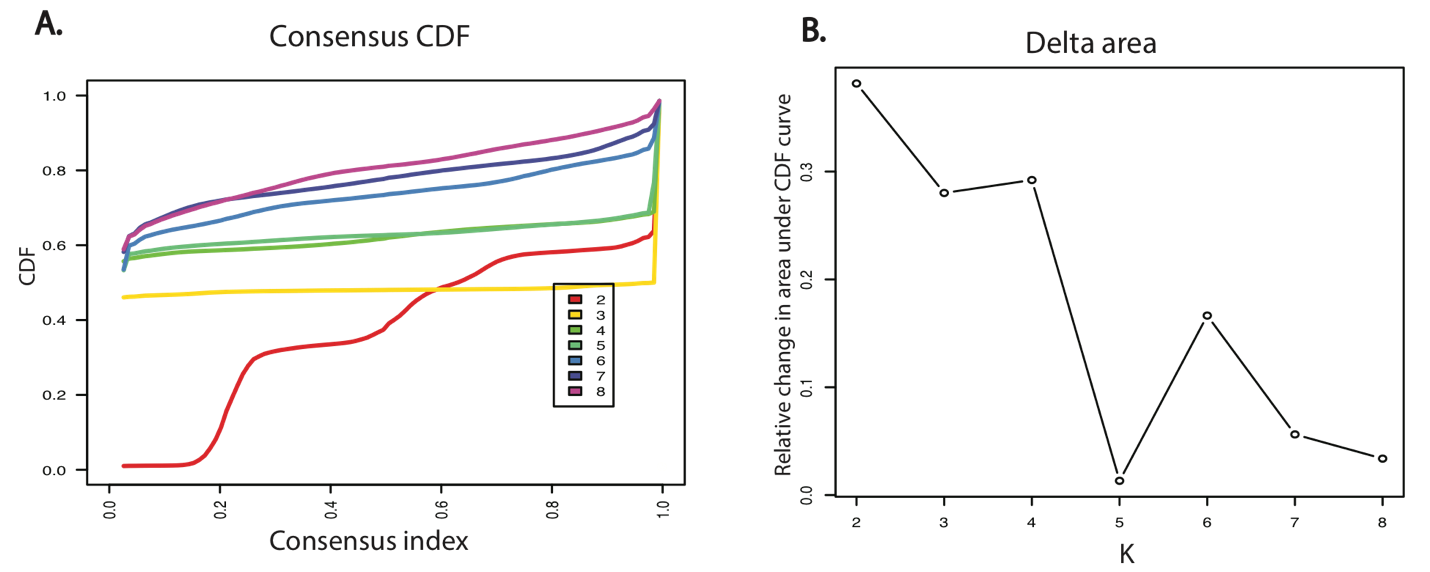
**Figure S1. The summary of the consensus cluster analysis.**

A. Consensus values heatmap, demonstrating a clearly delineated block structure for k=3, supporting a three-cluster solution. This is endorsed by the cumulative distribution function (CDF) curve, which approaches an ideal step function for k=3; B. The relative change in area under CDF curve illustrates that as k is increasing beyond k=3, there is a significant drop in the relative change in area under CDF curve, indicating an optimum at k=3.

**Figure S2. Anemia effects on 3-month death in ICH patients by age, sex, state of consciousness and severity.**

Solid boxes represent estimates of anemia effect; horizontal lines, 95% CI. Abbreviations: OR, odds ratio; CI, confidence interval; P, p value; GCS, Glasgow Coma Scale; NIHSS, National Institutes of Health Stroke Scale; BUN, blood urea nitrogen.

**Figure S3. Visualization of results from mediation effect of hematoma volume in different ICH subtypes.**

Unlike other ICH subtypes (A, B,C,D,F), in the systemic disease ICH subtype (E), the confidence interval of neither ACME or ADE crossed with the line of 0.00, suggesting a significant mediation effect of hematoma volume in systemic disease ICH subtype. HA, hypertensive angiopathy; CAA, cerebral amyloid angiopathy; ACME, average causal mediation effects; ADE, average direct effects.
